# AF1q inhibited T cell attachment to breast cancer cell by attenuating Intracellular Adhesion Molecule-1 expression

**DOI:** 10.20517/2394-4722.2018.84

**Published:** 2019-03-18

**Authors:** Jino Park, Jae Yeon Hwang, Alexandra Thore, Soojin Kim, Tomiteru Togano, Shotaro Hagiwara, Juw Won Park, William Tse

**Affiliations:** 1 James Graham Brown Cancer Center, University of Louisville School of Medicine, Louisville, KY 40202, USA.; 2 Division of Blood and Bone Marrow Transplantation, Department of Medicine, University of Louisville School of Medicine, Louisville, KY 40202, USA.; 3 Department of Computer Science and Computer Engineering, University of Louisville, Louisville, KY 40292, USA.; 4 Division of Haematology, National Center for Global Health and Medicine, Tokyo 162-8655, Japan.; 5 Division of Haematology, Tokyo Women’s Medical University, Tokyo 162-8666, Japan.

**Keywords:** MLLT11, AF1q, intercellular adhesion molecule-1, breast cancer, metastasis, RNA-sequencing

## Abstract

**Aim:**

To investigate whether AF1q, overexpressed in metastatic cells compared with the primary tumor cells, plays a pivotal role in breast cancer metastasis.

**Methods:**

To investigate whether AF1q has a responsibility in the acquisition of a metastatic phenotype, we performed RNA-sequencing (RNA-Seq) to identify the gene signature and applied the Metacore direct interactions network building algorithm with the top 40 amplicons of RNA-Seq.

**Results:**

Most genes were directly linked with intercellular adhesion molecule-1 (ICAM-1). Likewise, we identified that ICAM-1 expression is attenuated in metastatic cells compared to primary tumor cells. Moreover, overexpression of AF1q attenuated ICAM-1 expression, whereas suppression of AF1q elicited the opposite effect. AF1q had an effect on ICAM-1 promoter region and regulated its transcription. Decreased ICAM-1 expression affected the attachment of T cells to a breast cancer cell monolayer. We confirmed the finding by performing the analysis on Burkitt’s lymphoma.

**Conclusion:**

Attenuation of ICAM-1 by AF1q on tumor cells disadvantages host anti-tumor defenses through the trafficking of lymphocytes, which affects tumor progression and metastasis.

## INTRODUCTION

While primary lesion breast tumors are not fatal to the patient, the metastatic breast tumors are. For this reason, breast patients are often given other treatments after surgery to try to eliminate metastatic tumor cells that might have spread to other organs. Although the prognosis for breast cancer has improved during the last decade, we still lack specific treatments that target metastases. The discovery of such treatment will be of great benefit to patients.

The *AF1q* gene, located in chromosome 1, band 21, is an MLL fusion partner that was identified in acute myeloid leukemia patients with t (1; 11) (q21; q23) chromosomal abnormality^[1]^. The function of AF1q is not yet fully known; however, elevated AF1q expression is associated with poor clinical outcomes in various malignancies. We have demonstrated that the enhanced AF1q expression promotes cell proliferation, migration, sphere formation, and chemo-resistance *in vitro* and *in vivo* breast cancer models^[2]^. During the course of our study, we made an exciting discovery that AF1q physically interacts with TCF7, a key factor in the Wnt signaling pathway that enhances its activation^[2]^. The Wnt signaling pathway plays a leading role in various processes that are important for cancer progression, including cancer initiation, growth, cell death, differentiation, and metastasis^[3]^. Strikingly, we observed that AF1q-positive cancer cells are significantly more prevalent at metastatic sites than in primary breast tumors, when comparing paired samples from breast cancer patients^[2]^. Moreover, the Wnt signaling pathway was activated by AF1q crosstalk to the STAT3 pathway via the PDGF/PDGFR cascade^[4]^. The PDGF-B/PDGFR signaling cascade was activated upon enforced AF1q expression and this caused an increase in STAT3 DNA binding activity through *Src* kinase action in cancer cells^[4]^. Moreover, AF1q is one of the genes that are differentially expressed between highly metastatic breast cancer cells and their parent cells^[2]^. Importantly, when breast tumor cells were treated with doxorubicin or etoposide, endogenous AF1q expression was further activated, implying that current chemotherapeutics may increase the risk of metastasis of breast cancer. Taken together, characterization of AF1q-induced breast cancer and new treatment strategies for hyperactive AF1q expression patients are urgently needed.

Intercellular adhesion molecule-1 (ICAM-1) plays a key role in inflammatory conditions, nervous system development, and immune response through antigen recognition and lymphocyte surveillances^[5,6]^. There is still controversy regarding the contribution of ICAM-1 expression to tumor progression. Expression of ICAM-1 on tumor cells negatively correlates with tumor progression and development, including tumor size and lymph node metastasis^[7]^. A better prognosis was also reported for patients with ICAM-1 positive tumors compared with ICAM-1 negative tumors^[8,9]^. However, *in vitro* studies demonstrated that ICAM-1 overexpression can positively affect tumorigenicity^[10]^. Despite the controversy, it is clear that ICAM-1 plays a pivotal role in immune response. ICAM-1 levels on tumor cells stimulate T-cell receptor-mediated cellular immune response^[11]^. Recent studies revealed that ICAM-1 expression plays an important role in interactions between lymphokine-activated killer cells and cancer cells^[12,13]^. These results suggest that a decrease of ICAM-1 may be one of the mechanisms by which tumor cells escape cell-mediated cytotoxicity and lysis by the host cellular immune system.

Metastatic cancer cell, MDA-MB-231LN, is subtype of MDA-MB231 breast cancer cells and it has shown enhanced tumor growth and widespread metastasis than parents cell line in xenograft models^[14]^. We observed AFiq, a metastasis enhancer, is highly expressed in metastatic cancer cells (MDA-MB-231LN) than in the primary tumor cells (MDA-MB-231). In this study, we investigated whether AFiq is responsible in the acquisition of metastatic phenotype using RNA-sequencing (RNA-Seq) and applied the Metacore direct interactions network building algorithm. Intriguingly, most genes were directly linked with ICAM-1. Likewise, we identified that ICAM-1 expression is attenuated in metastatic cancer cells compared to primary cancer cells.

In addition to AF1q oncogenic functions, we demonstrated that intensity of AF1q expression in metastatic sites is higher than in primary sites^[2]^. Thus, similarly to that reported for certain oncogenes (i.e., Myc and Ras), AF1q has been shown to be endowed with a dual function in malignancy, being a protein apparently involved in both initiation and promotion of cancer progression through regulation of ICAM-1 expression. Our results suggest that targeting AF1q is valuable in developing treatments for breast cancer metastasis.

## METHODS

### Cell lines and cell culture conditions

MDA-MB-231 was purchased from American Type Culture Collection (ATCC). MDA-MB-231-luc-D3H2LN (MDA-MB-231LN) was purchased from Caliper Life Science. The cells were maintained as a monolayer culture in DMEM, supplemented with Glutamax and penicillin-streptomycin (Invitrogen). Fetal bovine serum (FBS) (Thermo Fisher Scientific) was added to the media. Burkitt’s lymphoma cell lines, Ramos, Akata, Mutu, Raji, Jiyoye, BL-5, BL-7, and BL-8, were maintained in RPMI-1640, supplemented with Glutamax, penicillin-streptomycin, and 10% FBS (Thermo Fisher Scientific).

Whole blood was collected from healthy volunteers at James Graham Brown Cancer Center, University of Louisville with donors’ written consent. The CD4-positive and CD8-positive human T cells were purified from buffy coats via positive selection using a 1:1 mixture of CD4- and CD8- MicroBeads (Miltenyi Biotec) according to the manufacturer’s protocol. Isolated T cells were maintained in RPMI-1640, supplemented with 300 IU/mL IL-2 (R&D) and 10% FBS (Thermo Fisher Scientific). All cells were maintained at 37 °C under a humidified atmosphere of 5% CO_2_.

### RNA-Seq

Total RNA were isolated from MDA-MB-231 and MDA-MB-231LN cells with the use of the Purelink RNA min Kit (Thermo Fisher Scientific) in triplicates. Of total RNA, 1 μg was depleted of cytoplasmic and mitochondrial ribosomal RNA using the Illumina Ribo-Zero Gold rRNA Removal Kit (Human/Mouse/Rat) (Illumina). The rRNA-depleted RNA was ligated with Illumina adapters and further processed for sequencing following the Illumina TruSeq Stranded Total RNA library preparation kit (Illumina). All samples were pooled and a 75-cycle single-end sequence run was performed using the Illumina High Output Kit v2 on the Illumina NextSeq 500 platform.

### RNA-Seq data analysis

RNA-Seq data were mapped using UCSC human genome, hg38, with STAR (version 2.5.2b). The read count and reads per kilobase of transcript per million mapped reads (RPKM) were calculated on the basis of the human GRCh38 Ensembl Release 91 gene annotation. To search differentially expressed genes, Cuffdiff (version 2.2.1) was used. For further analysis, 1485 significantly differentially expressed genes were selected as following criteria, when the value of averaged RPKM from three replicates is greater than or equal to 1 in at least one of the two samples, when the absolute value of log_2_ (fold-change) is greater than or equal to 1, and when false discovery rate is less than or equal to 0.01.

### Tumor data selection

We selected data from The Cancer Genome Atlas (TCGA) project to represent the breast invasive carcinoma (BRCA). We downloaded publicly available 1,222 RNA-Seq data of 1,092 breast cancer cases from TCGA database. Using FPKM values from all 1,222 samples, the expression level of AF1q was compared to that of ICAM-1.

### RT and real-time PCR analysis

Total RNA was subjected to reverse transcription (RT) with a High capacity RNA-to-cDNA kit (Thermo Fisher Scientific), and the resulting cDNA was subjected to real-time polymerase chain reaction (RT-qPCR) analysis with BrightGreen qPCR master mix (ABM) and a StepOne real time PCR system (Thermo Fisher Scientific). The amplification protocol comprised 40 cycles of incubations at 95 °C for 30 s and at 60 °C for 60 s. PCR primer sequences (forward and reverse) were as follows: 5’-TGAGTACAGCACCTTCAACTTC-3’ and 5’-GGGAAAGGAGTGGAAAGGAAG-3’ for AF1q; 5’-CAATGTGCTATTCAAACTGCCC-3’ and 5’-CAGCGTAGGGTAAGGTTCTTG-3’ for ICAM-1; 5’-CAGAGGGCTACAATGTGATGGC-3’ and 5’-GCTGAGGATTTGGAAAGGGTG-3’ for HPRT1.

### Plasmid construction

Full-length AF1q cDNA was inserted into pLUTdNB, which is a pTRIPZ base modified doxycycline-inducible vector by cloning the HindIII and XhoI fragments as previously described. Short hairpin RNA (shRNA) of AF1q was purchased from Open Biosystems. Empty pLUTdNB, and pTRIPZ-scramble were used as controls for pLUTdNB-AF1q, and pTRIPZ-AF1q shRNA, respectively.

### Viral production and infection

Lentivirus was produced by co-transfection of HEK 293T cells (ATCC) with the lentiviral constructs pVSV-G and psPAX2 (Addgene). Transfections were carried out using Lipofectamine 2000 (Invitrogen). For enforced AF1q expression or shRNA targeting, cells underwent lentiviral transduction and were selected using 1 μg/mL puromycin (Thermo Fisher Scientific) as previously described^[15]^. After antibiotic selection, cells were cultured in complete medium including 1 μg/mL doxycycline (Thermo Fisher Scientific) for AF1q or shRNA induction.

### Western blot

For blots of whole-cell lysates, cells were lysed directly in GLB buffer (2% SDS, 10% glycerol and 50 mmol/L Tris, pH 6.8), boiled, and separated by electrophoresis on a 4%−12% SDS-PAGE gradient gel. Proteins were transferred to PVDF membrane (Millipore) and blocked in 5% skim milk (Bio-Rad) in 0.05% PBST. Rabbit monoclonal anti-AF1q, ICAM-1, and β-actin antibodies were purchased from Abcam. After the appropriate antibody incubations, an enhanced chemiluminescence (Denville) system was used for developing blots.

### Reporter gene construct, transfection, and Luciferase assay

Dual-Luciferase reporter assays were performed using an ICAM-1 reporter plasmid. To construct reporter plasmids, 850 bases of an ICAM-1 promoter fragment were cloned to between the XhoI and HindIII sites of the pGL4-Luciferase reporter plasmid (Promega). Briefly, 1 × 10^4^ cells were plated in each well of a 96-well plate. The next day, the reporter plasmid (100 ng) and *Renilla* plasmid (10 ng, Promega) were co-transfected into the cells with lipofectamine 2000 (Thermo Fisher Scientific). After 48 h, cells were washed with PBS and assayed with the Dual-Luciferase reporter assay system (Promega) according to the manufacturer’s instructions. Luciferase activity was determined using Synergy H1 Multi-Mode reader (Biotek). Quantitation of luminescent signal from reporter plasmid was normalized by quantitation of the luminescent signal from *Renilla.*

### Surface and intracellular staining

Cells were harvested using non-enzymatic dissociation solution (Corning) and washed once with PBS. For staining of ICAM-1, cells were incubated in Human Fc Block (BD) for 10 min. Thereafter, cells were stained with FITC-conjugated antibody to ICAM-1 in the presence of Fc Block for 30 min at 4 °C. Cells were then washed 3× with PBS and analyzed by FACS Caliber (BD). Data analysis was performed using Flowjo V10 software (Ashland).

### Monocyte adhesion assay

Isolated T cells were incubated with Cell Tracker (Thermo Fisher Scientific) for 30 min at 37 °C and then 10^5^ cells per well were incubated on MDA-MB-231LN monolayers in HBSS supplemented with 2 mmol/L calcium and magnesium for 1 h. After removing nonadherent cells, the number of Cell Tracker-labeled cells bound to the MDA-MB-231LN cells was quantified under fluorescent microscopy.

### Immunohistochemistry

Tissue samples were fixed in 4% paraformaldehyde, embedded in paraffin, and then 4-μm sections were prepared. Sections were de-waxed and a steamer pre-treatment in Tris/EDTA buffer (DAKO) was performed. Endogenous peroxidase activity was quenched by incubation in 3% hydrogen peroxide in PBS. For blocking steps, avidin (Sigma-Aldrich), biotin (Sigma-Aldrich) in PBS, and a super block (IDlabs Biotechnology) were used. Rabbit monoclonal AF1q antibody and mouse monoclonal ICAM-1 antibody in a 1:200 dilution were incubated at 4 °C overnight. IHC detection was performed with the IDetect Super Stain System HRP (IDlabs Biotechnology). Specific signals were amplified using 3-amino-9-ethylcarbazole (IDlabs Biotechnology) under visual control, followed by counterstaining with hematoxylin.

### Statistical analysis

Quantitative data are presented as means ± SD and were subjected to analysis of variance followed by a *t* test with the use of Prism V.7 software (GraphPad Software). A *P* value of < 0.05 was considered statistically significant.

## RESULTS

### Next-generation sequencing and transcriptome data analysis

**Figure 1.**
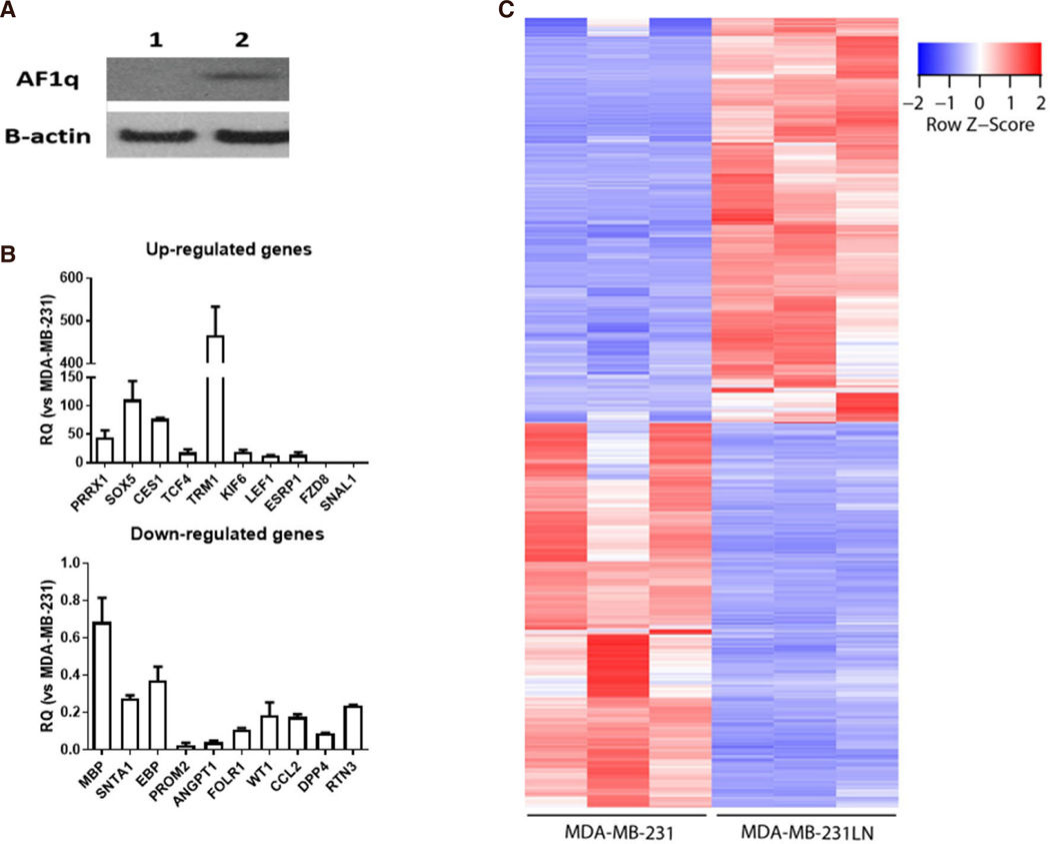
A: AF1q expression status in breast cancer cell lines (1: MDA-MB-231, 2: MDA-MB-231-luc2-LN); B: validation of relative expression of gene obtained from RNA-seq by qPCR. qPCR analyses were performed as described in the method section using randomly selected 20 genes (upregulated genes 10 and downregulated genes 10). Relative expression values are presented as an average ± SD of three biological replicates; C: significantly expressed genes. 1,485 genes are significantly expressed in both cell lines with three biological replicates, respectively. Among those, transcripts of 762 genes increased in MDA-MB-231LN cell line and those of 723 genes decreased

We observed AF1q, a metastasis enhancer, was highly expressed in metastatic cells, MDA-MB-231LN (invasive subline from MDA-MB-231), than in the primary tumor cells (MDA-MB-231) [Figure 1A]. To validate the results of RNA-seq, we performed RT-qPCR analysis using randomly selected genes [Figure 1B]. Both MDA-MB-231 and MDA-MB-231LN cell lines were subjected to RNA-Seq analysis with three biological replicates for each cell line. The 75 bp-long single-end sequencing reads were mapped to the human genomic reference using STAR aligner. More than 27.7 million sequencing reads were mapped to genome on each replicate (averaging 35.3 million mapped reads) [Supplementary Table 1]. Out of the 58,289 annotated genes, 18,042 (31.0%) by Ensemble were expressed with more than 10 read counts on at least one of our replicates [Supplementary Table 2]. Only uniquely mapped reads were used further for searching differentially expressed genes using Cuffdiff. Total 1,485 genes were selected as significantly differentially expressed with the criteria explained above. Among those, 762 genes showed increased value of RPKM in MDA-MB-231LN cell line compared to MDA-MB-231 and 723 genes showed the opposite. Row-scaled RPKM value of each replicate represents that the differential expression of genes is consistent throughout replicates of each cell line [Figure 1C].

**Figure 2.**
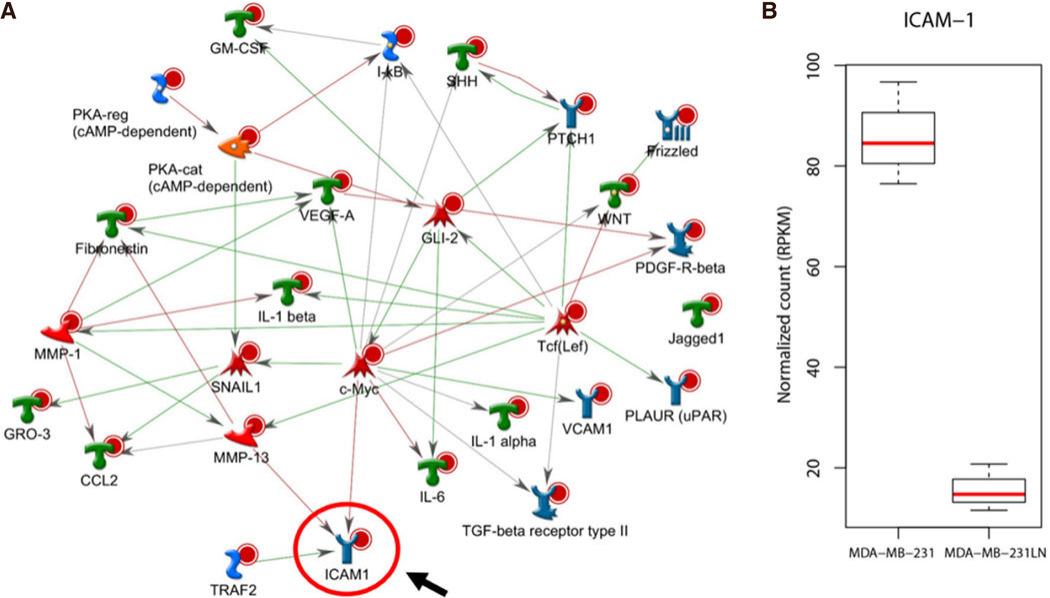
Functional interaction network analysis. A: 1,485 significantly selected genes were further analyzed for pathway process using Metacore. Top 40 network objects were, then, used for building direct interactions. ICAM-1 is directly linked with most genes and positioned at the end of the pathways. Red line represents suppression, blue line represents activation; B: RNA-seq shows that expression level of ICAM-1 is drastically decreased in MDA-MB-231LN cell line compared to MDA-MB-231

With those differentially expressed genes, pathway analysis was performed using Metacore. By selecting top 40 network objects resulted from the pathway analysis [Table 1], direct interaction network building algorithm was applied. Interestingly, pathway process showed that most genes were directly linked with ICAM-1 [Figure 2A]. In addition, we found out that ICAM-1 transcript level was significantly decreased in MDA-MB-231LN cell line compared to MDA-MB-231 confirming that ICAM-1 expression is attenuated in metastatic cell line [Figure 2B].

To explore the relationship of AF1q with ICAM-1 in breast cancer, we used 1,222 RNA-seq data of 1,092 breast cancer cases from TCGA database. Using FPKM values from all 1,222 samples, the expression level of AF1q was compared to that of ICAM-1. Vast majority of the samples did not show clear correlation between the expression levels of AF1q and ICAM-1 [Supplementary Figure 1]. Interestingly, however, all those with high FPKM values of AF1q showed low expression level of ICAM-1 supporting our observation that overexpression of AF1q attenuated ICAM-1 expression. Although majority of samples did not show clear correlation, this finding is consistent with our previous report that the role of AF1q is a co-factor rather than a transcription factor^[2]^.

### ICAM-1 is transcriptionally regulated by AF1q

**Figure 3.**
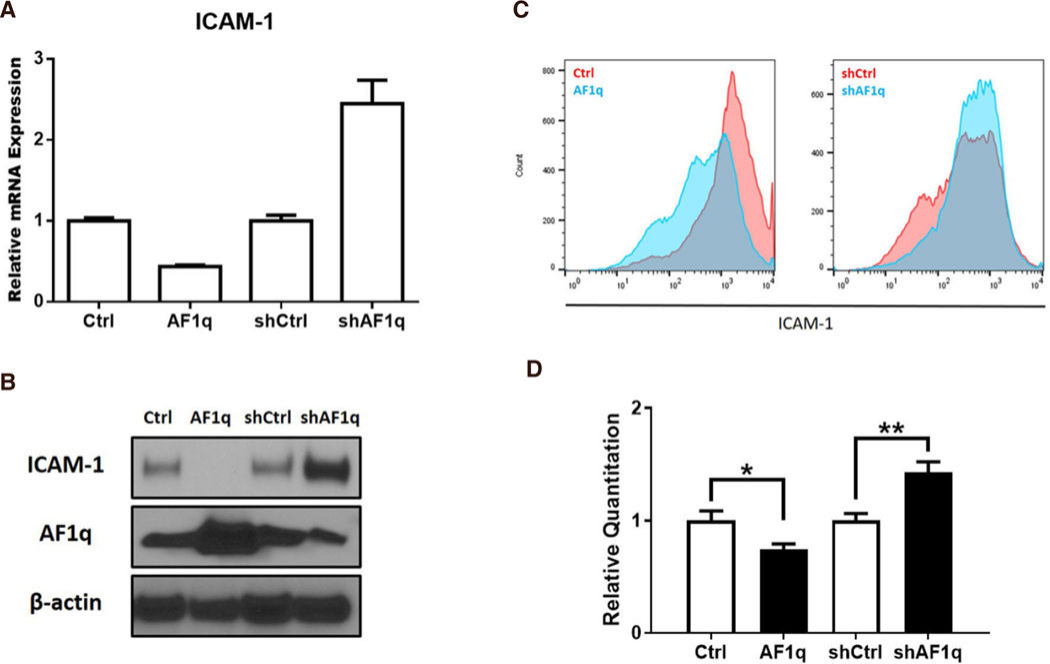
Transcriptional regulation of ICAM-1 expression by AF1q in breast cancer. A: ICAM-1 mRNA expression was quantified using qPCR in MDA-MB-231LN cells engineered to overexpress or suppress AF1q; B: the blot shows ICAM-1 protein expression; C: MDA-MB-231LN cells were stained with ICAM-1 mAb and flow cytometry analysis were performed using FACS Calibur. On left panel, Red is profile of the MDA-MB-231LN/Ctrl and Blue is MDA-MB-231LN/AF1q. On the right panel, Red is MDA-MB-231LN/shCtrl and Blue is shAF1q cells. Representative data of three similar experiments are shown; D: Luciferase activity in HEK293T cells transfected with reporter constructs of the proximal promoter of ICAM-1. Renillar Luciferase activity was used to normalize the promoter activity

To demonstrate that the AF1q expression was involved in ICAM-1 gene expression in breast cancer, we first experimentally overexpressed or suppressed AF1q expression in MDA-MB-231LN. We used a lentiviral transduction system to overexpress and suppress AF1q with endogenous AF1q expression. As shown in Figure 3A and B, overexpressed AF1q (AF1q) remarkably decreased ICAM-1 mRNA and protein expression, compared to that of control (Ctrl). The ICAM-1 expression, however, was increased by the suppression of AF1q with shRNA (shAF1q) than control (shCtrl). These results indicate that AF1q regulates ICAM-1 expression in transcription. FACS analysis comparing ICAM-1 surface expression on cells show that the attenuation of ICAM-1 on the surface of MDA-MB-231LN in response to AF1q was also confirmed [Figure 3C].

We assessed whether AF1q influences ICAM-1 promoter activity by performing a Luciferase reporter assay. We first experimentally overexpressed or suppressed AF1q expression in HEK293, then, HEK293 cells with a construct containing 850 bases of an ICAM-1 promoter fragment. Overexpressed AF1q displayed 0.7 fold lower luminescence. However, the luminescence was 1.4 fold higher when AF1q expression was suppressed by shAF1q [Figure 3D].

### ICAM-1 plays an important role in T cell adhesion and cytolysis

**Figure 4.**
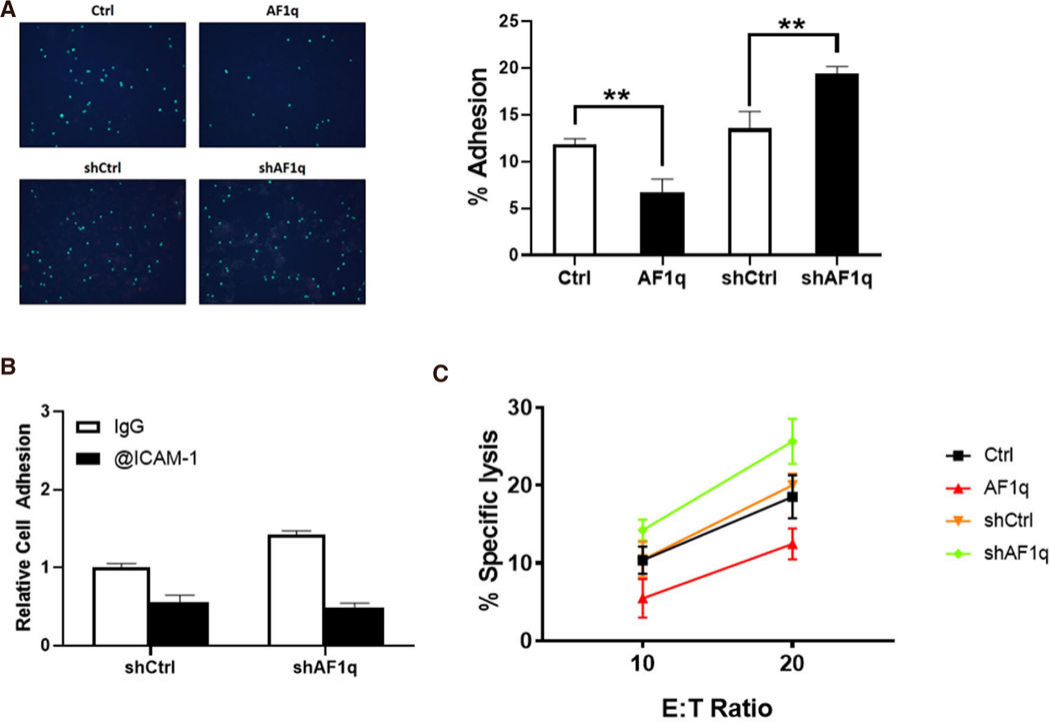
ICAM-1 is important for attachment and cytotoxicity of T cells to breast cancer. A: Labelled T cells attached to MDA-MB-231LN cells monolayer. AF1q significantly reduced the number of attached T cells to the monolayer. When AF1q expression was suppressed, the number of attached T cells was increased. The Bar graph represents the % adhesion cells of total cells; B: ICAM-1 blocking antibody significantly reduced the number of attached T cells to the monolayer; C: a 72 h cytotoxicity assay was performed in 24 well plates where co-cultured at 10:1 or 20:1 E:T ratio with MDA-MB-231LN cells

Because ICAM-1 is associated with the recognition of T cells, we wanted to determine whether ICAM-1 dysregulation by AF1q is essential for the T cell attachment to breast cancer cells. To demonstrate that ICAM-1 plays a critical role in the adhesion of T cells, we performed an *in vitro* adhesion assay. The attachment between breast cancer cell monolayer and T cells was significantly reduced by AF1q-induced ICAM-1 attenuation, while enhanced ICAM-i expression by AF1q suppression increased the attachment [Figure 4A]. The attachment between T cells and breast cancer cell monolayer was significantly reduced by pretreatment with a blocking antibody to ICAM-1 [Figure 4B].

Cell cytotoxicity assays are shown in Figure 4C where the corresponding MDA-MB-231LN sublines were used as the target cell. Assays were carried out using *ex vivo* expanded T cells derived from healthy donors. These data indicate that ICAM-1 can function as an important determinant of a tumor cell’s sensitivity to T cell-mediating killing.

### AF1q reciprocally regulate expression of ICAM-1

**Figure 5.**
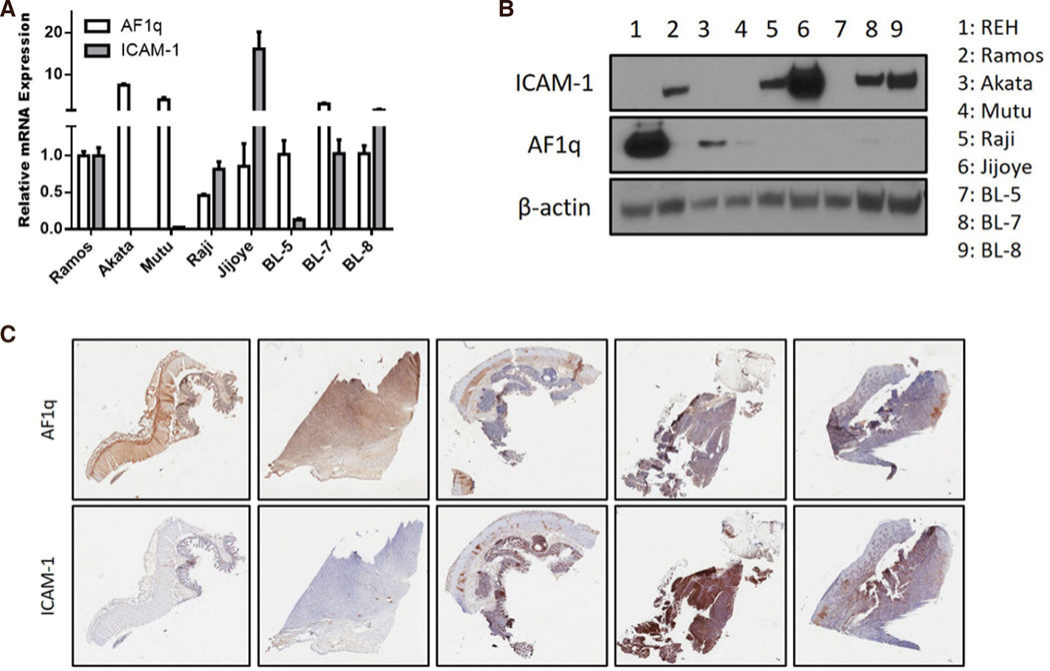
AF1q reciprocally regulates the expression of ICAM-1 in Burkitt’s lymphoma. A: ICAM-1 mRNA expression was quantified using qPCR in MDA-MB-231LN cells engineered to overexpress or suppress AF1q; B: Western blot analysis of ICAM-1 and AF1q in Burkitt’s lymphoma cell lines; C: representative images of AF1q and ICAM-1 staining in human Burkitt’s lymphoma tumor metastasis tissues. High IHC staining of AF1q in tissue samples show low ICAM-1 expression

We extended our finding to Burkitt’s lymphoma. It is well known that downregulation of ICAM-1 in Burkitt’s lymphoma enhances the probability of escape of tumor cells from T cell surveillance^[16]^. RT-qPCR and Western blot analysis showed that the AF1q expression at both the mRNA and protein levels reciprocally regulated the expression of ICAM-1 in Burkitt’s lymphoma cell lines [Figure 5A and B]. We validated our finding in Burkitt’s lymphoma patient samples. To investigate the expression levels of AF1q and ICAM-1 in Burkitt’s lymphoma patient samples, immunohistochemical staining of patient tissues was performed. We have observed identical results from the IHC study [Figure 5C]. These findings are consistent with observation in breast cancer cells.

## DISCUSSION

We have here identified ICAM-1 which reciprocally regulated by AF1q was associated with metastasis of cancer cells. Although elevated AF1q expression is associated with poor clinical outcomes in various malignancies^[4–6]^, the function of AF1q is not yet fully understood. Our findings further explain why high AF1q expression is associated with poor clinical outcomes. However, greater mechanistic understanding of how AF1q is involved in promoting cancer metastasis is necessary before these laboratory investigations can be translated into clinical interventions. Thus, it is very important to continue to investigate the underlying biological functions of AF1q and its association with breast cancer metastasis.

We observed that nuclear factor-kappa B (NF-κB) activity was attenuated in response to AF1q expression in breast cancer cells (data not shown). NF-κB translocated to the nuclers binds to the proximal NF-κB consensus sequence of the ICAM-1 promoter and binding of NF-κB to the proximal binding site of the ICAM-1 promoter induces transcriptional activity^[17]^. Published reports show that Wnt/β-catenin negatively regulates NF-κB activity through a β-catenin-NF-κB interaction in colon and breast cancer cells^[18,19]^. β-catenin can physically complex with NF-κB, resulting in a reduction of NF-κB DNA binding, transactivation activity, and target gene expression^[20]^. Activated β-catenin is associated with repressed NF-κB activity in human cancer cells^[20]^. Interestingly, the interaction between them is only indirect, as these two proteins do not bind to each other without a helper protein. Structurally, AF1q has highly acidic peptide regions highly conserved between species that fulfill the criteria for an acidic blob, a typical feature for cofactors. We also noted an internal peptide repeat within the sequence of 90 amino acids of human AF1q, which is located at both ends of the peptide, indicative of similar binding interfaces^[2]^. Previously, we reported that AF1q enhances the TCF7/LEF/β-catenin complex binding affinity as a cofactor. When we performed immunoprecipitation with NF-κB antibody in cancer cells overexpressing AF1q, we observed that β-catenin and AF1q were pulled down together (data not shown). However, it is not clear yet whether AFiq promotes protein interaction between β-catenin and NF-κB. These results suggest that activated β-catenin by AF1q would archive higher affinity to bind with NF-κB.

Cancer cells utilize multiple mechanisms to prevent host immune cells from exercising their antitumor activities. Many of these mechanisms are now known on a cellular and molecular level. These mechanisms, which enable the tumor to escape from the host immune system and to progress, are being intensively investigated in hope of finding therapeutically safe and effective inhibitors able to counteract tumor-induced immunosuppression. Tumor escape has been a major problem in cancer immunotherapy, and it has been held responsible for the failure of many immune interventions in cancer. For this reason, it is important to study and understand the various suppressive pathways human tumors utilize.

Tumors use blood vessels to supply themselves with oxygen and nutrients as well as for waste removal^[21]^. Lymphocytes also use blood vessels as the gateway where intergrin interactions with endothelial cell adhesion molecules are required to infiltrate into the tumor^[22,23]^. The downregulation of ICAM-1 by several inhibitory mechanisms limiting T cell transendothelial migration have been described^[24–26]^. Also, other componets of the tumor stroma and cancer-associated fibroblasts, can suppress T cell infiltration, which can influence cancer progression and metastasis^[27]^.

The interction between tumor and lymphocytes through ICAM-1 plays an important role in leukocyte adhesion, transduction, and cytolysis ^[28–30]^. For example, tumor clones from melanoma metastasis split into 2 groups with high and low susceptibility to killing by IL-2 activated lymphocytes. The subset with low lysablity expressed ICAM-1 at levels 10 fold lower than those of tumor clones with high lysability^[31]^. These results suggest that a constitutively high expression of ICAM-1 on tumors would be the parameter contributing to the high lysability of these tumor cells by any effector.

This study investigated the role of AF1q-attenuated ICAM-1 in progression and metastasis of breast cancer. Based on published reports, ICAM-1 strongly stimulates metastasis but also regulates lymphocyte infiltration via interactions between immune cells and malignant cells. This suggests that ICAM-1 needs an on-off switch for cancer progression and metastasis. It needs “off” to escape from host immune surveillances system in the initial phase of cancer, but “on” to invade and grow afterwards. Our results suggest that AF1q is a switch for ICAM-1 expression. Therefore, AF1q is a promising target for developing treatment for breast cancer metastasis.

## Supplementary Material

Supplementary Figure S1

Supplementary Table S1

Supplementary Table S2

## Figures and Tables

**Table 1.** Top 40 network objects ranked by occurrence in top 50 pathway maps

Ranking	Network objects	Number of maps containing network object	Ranking	Network objects	Number of maps containing network object

1	IL-6	24	10	TGF-β receptor type II	8
2	ICAM-1	20	11	NFKBIA	7
3	IL-1 beta	18	11	MHC class II	7
4	p38 MARK	16	12	WNT5A	6
5	VCAM-1	15	12	GRO-2	6
6	VEGF-A	12	12	Tcf(Lef)	6
6	c-Myc	12	12	Bcl-XL	6
7	GM-CSF	11	12	WNT	6
7	CCL2	11	13	GRO-3	5
8	I-kB	10	13	PLAUR	5
8	PTCH1	10	13	IL-1 α	5
8	SHH	10	13	SNAIL1	5
9	MMP-1	9	13	PKA-cat	5
10	Fibronectin	8	13	MMP-13	5
10	TCF7L2	8	13	PDGF-R-β	5
10	Frizzled	8	13	CSF1	5
10	TLR2	8	13	PKA-reg	5
10	GLI-2	8	14	Jagged1	4
10	Lef-1	8	14	TRF2	4

Network object ranking reflects the popularity of each network object in pathway map. Smaller rank value means that network object occurs in greater number of maps. ICAM-1: intercellular adhesion molecule-1
