# Supplementary material for: AF1q inhibited T cell attachment to breast cancer cell by attenuating Intracellular Adhesion Molecule-1 expression
Source: J Cancer Metastasis Treat. Author manuscript; Available in PMC 2019 Jul 11. (PMC6623974; doi:10.20517/2394-4722.2018.84)
Supplement: Supplementary Figure S1 [file NIHMS1018943-supplement-Supplementary_Figure_S1.pptx]

## Slide 1
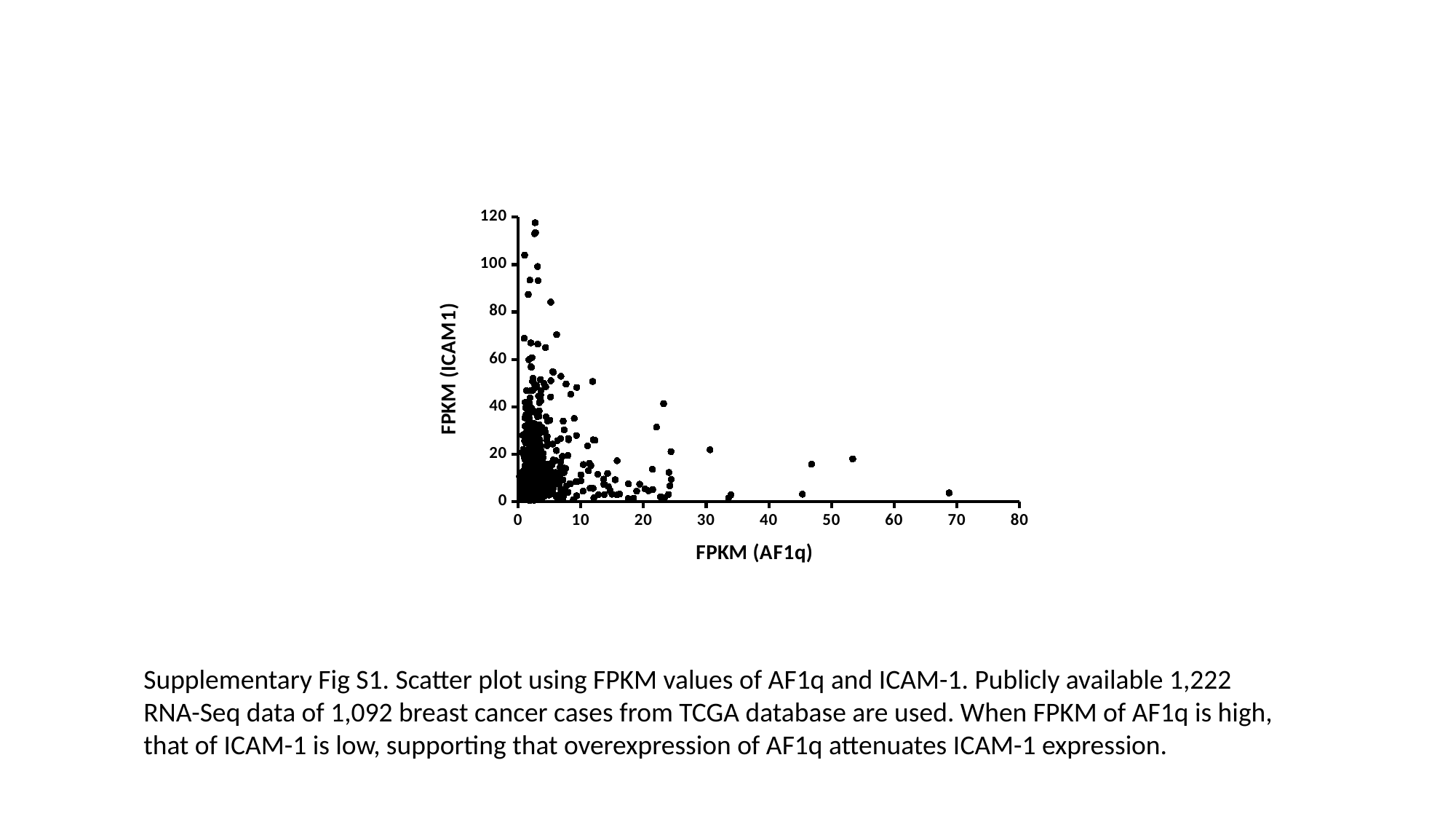

### Chart
| Category | ICAM1 |
|---|---|Supplementary Fig S1. Scatter plot using FPKM values of AF1q and ICAM-1. Publicly available 1,222 RNA-Seq data of 1,092 breast cancer cases from TCGA database are used. When FPKM of AF1q is high, that of ICAM-1 is low, supporting that overexpression of AF1q attenuates ICAM-1 expression.
