# Supplementary material for: AF1q inhibited T cell attachment to breast cancer cell by attenuating Intracellular Adhesion Molecule-1 expression
Source: J Cancer Metastasis Treat. Author manuscript; Available in PMC 2019 Jul 11. (PMC6623974; doi:10.20517/2394-4722.2018.84)
Supplement: Supplementary Table S1 [file NIHMS1018943-supplement-Supplementary_Table_S1.docx]

Supplementary table 1. The summary of sequence reads from RNA sequencing analysis

| **Sample** | **Total Reads** | **Mapped Reads** |
| --- | --- | --- |
| MDA-MB-231 #1 | 46,033,181 | 30,912,171 |
| MDA-MB-231 #2 | 38,871,900 | 37,383,121 |
| MDA-MB-231 #3 | 43,277,345 | 41,694,766 |
| MDA-MB-231LN #1 | 37,155,092 | 27,742,980 |
| MDA-MB-231LN #2 | 48,253,014 | 38,148,425 |
| MDA-MB-231LN #3 | 37,489,646 | 36,041,713 |
